# Biological relevance of alternative splicing in hematologic malignancies

**DOI:** 10.1186/s10020-024-00839-2

**Published:** 2024-05-17

**Authors:** Monika Szelest, Krzysztof Giannopoulos

**Affiliations:** Department of Experimental Hematooncology, Medical University of Lublin, Chodzki 1, 20-093 Lublin, Poland

**Keywords:** Splicing, Leukemia, Splicing factors

## Abstract

Alternative splicing (AS) is a strictly regulated process that generates multiple mRNA variants from a single gene, thus contributing to proteome diversity. Transcriptome-wide sequencing studies revealed networks of functionally coordinated splicing events, which produce isoforms with distinct or even opposing functions. To date, several mechanisms of AS are deregulated in leukemic cells, mainly due to mutations in splicing and/or epigenetic regulators and altered expression of splicing factors (SFs). In this review, we discuss aberrant splicing events induced by mutations affecting SFs (*SF3B1*, *U2AF1*, *SRSR2*, and *ZRSR2*), spliceosome components (*PRPF8*, *LUC7L2*, *DDX41,* and *HNRNPH1*), and epigenetic modulators (*IDH1* and *IDH2*). Finally, we provide an extensive overview of the biological relevance of aberrant isoforms of genes involved in the regulation of apoptosis (e. g. *BCL-X*, *MCL-1*, *FAS*, and *c-FLIP*), activation of key cellular signaling pathways (*CASP8*, *MAP3K7*, and *NOTCH2*), and cell metabolism (*PKM*).

## Background

Alternative splicing is a dynamic process in which the primary gene transcripts (pre-mRNAs) undergo splicing at distinct splice sites, and internal sequences are selectively removed, while the coding sequences are joined together to generate a different number of mature mRNA splicing variants. AS provides the expression of specific isoforms in a developmental and tissue-specific manner, thus maintaining cellular homeostasis. Differential use of splice sites contributes to proteome diversity, as over 95% of human genes were found to undergo some splicing event. AS events mediate the selective degradation of mRNA by introducing the premature termination codons (PTC) to mature mRNA, thereby activating the process of nonsense-mediated mRNA decay (NMD) (Darman et al. 2015). Notably, AS influences mRNA localization, stability, access to regulators, and translation efficiency, as it can modify the untranslated regions (UTRs) (Steri et al. 2018).

**Fig. 1:**
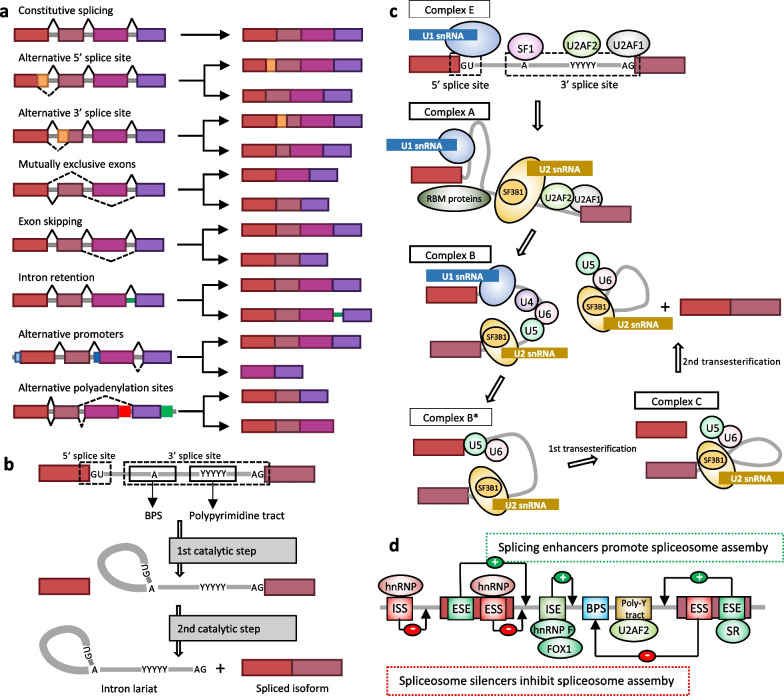
Types of alternative splicing (AS) and splicing regulation. **A** Distinct types of AS. **B** Two transesterification reactions resulting in the removal of intron and joining together of exons. **C** Spliceosome assembly. **D** The cooperation of *trans-* and *cis*-regulatory elements in the regulation of AS. BPS, branch point sequence; ISS, intronic splicing silencer; ESE, exonic splicing enhancer; ESS, exonic splicing silencer; ISE, intronic splicing enhancer

Diverse mechanisms are involved in production of different transcript isoforms in human cells. These events include: (1) alternative 5′ or 3′ splice site selection, which leads either to the retention of a restricted intronic sequence or exclusion of smaller exon; (2) mutually exclusive exons—distinct exons are combined to generate different transcript isoforms, but never coincide in the same isoform; (3) exon skipping, which causes the exclusion of a selected exon from the mature mRNA; (4) intron retention—the entire intronic region is retained in the mature mRNA; (5) transcription factor-mediated alternative promoter selection—distinct promoters of RNA polymerase II are used, thus affecting splice site choice; and (6) alternative sites of polyadenylation—different polyadenylation sites are chosen to produce alternative 3′-ends (Fig. 1A). Importantly, if the splice event provides the restored open reading frame, produced splice variants would encode protein isoforms with distinct functional and structural features.

Both constitutive and AS is tightly controlled by the spliceosome, a complex structure composed of five small nuclear ribonucleoprotein particles (snRNPs: U1, U2, U4, U5, U4/U6) and numerous auxiliary proteins, which recognize splice sites and carries out the two splicing reactions (Fig. 1B). The accurate recognition of the core sequences—branch point sequence (BPS) together with 5′ and 3′ splice sites—warrant that spliceosome assembly occurs properly. The spliceosome assembly starts with the recognition of 5′ splice site by the U1 snRNP and binding of the splicing factor 1 (SF1), the U2 auxiliary factor 2 (U2AF2), and U2AF1 to the BPS, the polypyrimidine tract, and the 3′ splice site AG dinucleotide, respectively, resulting in the formation of the complex E. This step is followed by the ATP-dependent replacement of the SF1 by U2 snRNP component SF3B1 at the BPS, leading to the formation of the pre-spliceosomal complex A. The subsequent recruitment of the U4/U6-U5 tri-snRNP complex forms complex B, which undergoes conformational changes and remodeling, leading to the formation of the catalytically active complex C that excises the intron and joins the exons together via two transesterification reactions (Fig. 1C) (Black et al. 2023).

As any error during the pre-mRNA splicing might lead to the formation of an improper transcript, a large number of protein regulators (*trans*-acting elements), which interact with each other and with specific RNA sequence elements (*cis*-regulatory factors), control the spliceosome machinery (Fig. 1D). *Cis*-acting elements are short nucleotide sequences divided into four categories, determined by their location and function: exonic splicing enhancers (ESEs), exonic splicing silencers (ESSs), intronic splicing enhancers (ISEs), and intronic splicing silencers (ISSs). Thus, *trans*-acting RNA binding proteins (RBPs) and SFs regulate the splicing process via binding to the specific intronic and/or exonic enhancing/silencing motifs. Recruitment of RBPs to ESE and ISE secures the proper formation of the spliceosome and provides splice site selection and retention of an exon. RNA recognition motifs of ESE are mainly bounded by the SR (Ser-Arg) proteins, while ISEs stimulate splicing via interaction with RBFOX1, RBFOX2, heterogenous nuclear RNP (hnRNP) F and hnRNP H. Conversely, RPBs binding to exonic or intronic silencing motifs (ESS or ISS, respectively) inhibits spliceosome assembling and stimulates exclusion of an exon (Dvinge and Bradley 2015). Furthermore, AS is influenced by RNA secondary structure, as it controls the ability of RBPs to bind specific motifs in pre-mRNA (Bartys et al. 2019).

## AS in hematological malignancies

Although mechanisms of AS are similar to constitutive splicing, various features impact the process of the splice site selection. While abnormal transcripts are usually degraded, the dysfunctional elements of splicing machinery might cause the accumulation of inaccurate splice variants in different cell compartments. Thus, the disruption of the mechanism of AS might result in decreased levels of normal proteins or an imbalance in the quantitative ratios among tissue-specific isoforms.

To date, over 70% of SFs are differentially expressed in cancer cells (Sveen et al. 2015). In comparison to non-malignant tissues, tumor cells exhibit up to 30% more AS events (Lehmann et al. 2018). Functional studies revealed that specific cancer-related splicing events affect protein domains that are also often mutated in tumors, leading to disruption of interactions between proteins involved in key signaling pathways in cells (Climente-González et al. 2017). Moreover, a study including 16 types of cancer showed global intron retention, which was presented in tumor cells even lacking mutations of splicing machinery elements (Dvinge and Bradley 2015). However, there is a large variability in intron-retaining mechanisms among analyzed cancer types. Regarding hematological malignancies, the production of aberrantly spliced isoforms was reported to contribute to the acquisition of drug resistance (Berman et al. 2016; Sotillo et al. 2015). Therefore, AS might provide an important source of novel therapeutic targets and cancer biomarkers.

**Fig. 2:**
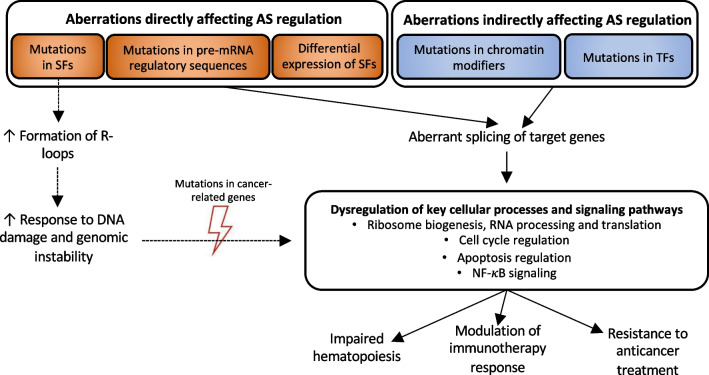
Aberrant regulation of alternative splicing (AS) and its contribution to the pathogenesis of hematologic malignancies. Recent evidence indicates that early spliceosome mutations that affects pre-spliceosome assembly might disrupt transcription, which, in turn, promotes R-loop formation. However, cell division kinetics might have a particular relevance for the consequences of DNA:RNA hybrid formation, as rapidly dividing cells display relatively high sensitivity for R-loop-related DNA damage, probably due to lack of time for DNA repair. In turn, damaged DNA that is not sufficiently repaired may lead to genomic instability, resulting in cumulative mutation burden over a few divisions. On the other hand, distinct hematological SF-mutated malignancies do not display genomic instability, thus the exact mechanism of transcription disruption and abundant R-loop formation by distinct spliceosome-related mutations, as well as interplay with AS events should be investigated

Emerging evidence indicates that AS aberrations might contribute to the leukemic transformation, cancer progression and response to treatment (Fig. 2).

To date, several mechanisms of AS are deregulated in leukemic cells, mainly due to mutations in splicing and/or epigenetic regulators and altered expression of SFs (Crews et al. 2016; Black et al. 2018; Fei et al. 2018). For instance, studies on AS in diffuse large B-cell lymphoma (DLBCL) revealed that the exon expression profile enables patient stratification to molecular-specific subgroups better than gene-level expression profiles (Leivonen et al. 2017). Interestingly, the highest frequency of AS events was observed in acute myeloid leukemia (AML) samples relative to control normal cells (Dvinge and Bradley 2015). A genome-wide AS screening of patients with AML showed that about 29% expressed genes were differentially spliced in comparison to healthy donors CD34 + progenitor cells (Adamia et al. 2014a).

Recently, a comprehensive transcriptome analysis revealed a widespread change in SFs expression and AS in therapy-resistant secondary AML stem cells and myelodysplastic syndrome (MDS) progenitors (Crews et al. 2016). It was reported that expression of an aberrantly spliced variant of a membrane antigen CD20 in B cell lymphomas generates immunogenic epitopes, which are recognized by T lymphocytes, therefore resulting in the killing of autologous lymphoma B cells (Vauchy et al. 2015). Furthermore, a proteomics study by Johnston et al. (Johnston et al. 2018) revealed a subtype-independent protein expression profile in patients with chronic lymphocytic leukemia (CLL).

## Splicing-related mutations in hematological malignancies: splicing factors

To date, numerous somatic mutations of genes involved in the spliceosomal machinery have been reported in patients with leukemia, among which those affecting *SF3B1*, *U2AF1*, *SRSF2*, and *ZRSR2* are the most common (Table 1).

**Table 1:** Recurrently mutated SFs in hematological malignancies and effect on prognosis

Hematologic neoplasm	SF	SF mutational frequency	Clinical outcome
CLL	*SF3B1*	5–31% Quesada et al. (2011); Oscier et al. (2013); Leeksma et al. (2019); Brown et al. (2018); Nadeu et al. (2016)	• Shorter TTT Quesada et al. (2011); Nadeu et al. (2016); Jeromin et al. (2014) • Shorter OS Quesada et al. (2011); Zhang et al. (2017) • Shorter PFS and OS Zhang et al. (2017) • No impact on PFS and ORR Brown et al. (2018)
	U1 snRNA	3.8% Shuai et al. (2019)	• No effect on OS, shorter TTT Shuai et al. (2019)
MDS	*SF3B1*	7–81% Malcovati et al. (2015, 2020); Jafari et al. (2020)	• Longer EFS Papaemmanuil et al. (2011) • Longer OS Malcovati et al. (2015, 2020); Gangat et al. (2018); Komrokji et al. (2015) • Lover risk of evolution into AML Malcovati et al. (2015, 2020) • The absence of *SF3B1*^K700E^ independently predicted worse OS Kanagal-Shamanna et al. (2021) • No impact on OS Jafari et al. (2020); Thol et al. (2012)
	*SRSF2*	4–18% Arbab Jafari et al. (2018)	• Shorter OS, higher risk of evolution into AML Thol et al. (2012); Zheng et al. (2017) • Shorter OS Arbab Jafari et al. (2018)
	*U2AF1*	7.5–17% Li et al. (2018); Graubert et al. (2011); Wu et al. (2013)	• Shorter OS, shorter TTL in younger (< 50 years old) and lower-risk patients Wu et al. (2013) • Shorter OS, higher risk of secondary AML Wang et al. (2019a) • Shorter OS Li et al. (2018) • No impact on OS Thol et al. (2012)
	*ZRSR2*	3–7% Thol et al. (2012); Haferlach et al. (2014)	• No effect on OS Thol et al. (2012)
MDS with RS	*SF3B1*	16–77% Migdady et al. (2018); Mangaonkar et al. (2018)	• Longer OS Migdady et al. (2018); Mangaonkar et al. (2018) and LFS Migdady et al. (2018)
MDS without RS	*SRSF2*	10.1% Kang et al. (2015)	• Shorter PFS Kang et al. (2015)
	*U2AF1*	7.8% Kang et al. (2015)	• Shorter PFS Kang et al. (2015)
	*SF3B1*	7% Kang et al. (2015)	• No impact on AML transformation, PFS and OS Kang et al. (2015)
De novo AML	*SRSF2*	5.4% Hou et al. (2016)	• Lower CR rate, shorter OS, trend of shorter DFS Hou et al. (2016)
	*U2AF1*	3% Hou et al. (2016)	• Lower CR rate, shorter OS and DFS Hou et al. (2016)
	*SF3B1*	2.4% Hou et al. (2016)	
Secondary AML	*SRSF2*	16–18.9% Venton et al. (2018); Zhang et al. (2012)	• Shorter OS Venton et al. (2018) • No impact on EFS or OS, or CIR in AML patients who received an allogenic HSCT Grimm et al. (2021)
Primary myelofibrosis	*SRSF2*	17–18% Lasho et al. (2012a); Tefferi et al. (2016)	• Shorter OS and LFS (Lasho et al. (2012a); Tefferi et al. (2016)
	*U2AF1*	16% Tefferi et al. (2018)	• No impact on LFS, shorter OS in cases with *U2AF1*^Q157^ mutation Tefferi et al. (2018)
	*SF3B1*	6.5% Lasho et al. (2012b)	• No impact on OS Lasho et al. (2012b)
CMML	*SRSF2*	25–47% Arbab Jafari et al. (2018)	• No impact on OS Arbab Jafari et al. (2018); Duchmann et al. (2018)
MCL	*HNRNPH1*	10% Pararajalingam et al. (2020)	• Shorter OS and PFS Pararajalingam et al. (2020)

SF, splicing factor; TTT, time to treatment; PFS, progression-free survival; ORR, overall response rates; OS, overall survival; MDS, myelodysplastic syndrome; EFS, event-free survival; AML, acute myeloid leukemia; TLL, time to leukemia transformation; RS, ring sideroblast; LFS, leukemia-free survival; CR, complete remission; DFS, disease-free survival; CIR, cumulative incidence of relapse; CMML, chronic myelomonocytic leukemia; MCL, mantle cell lymphoma; BPDCN, blastic plasmacytoid dendritic cell neoplasm

While *SF3B1*, *U2AF1*, and *SRSF2* are affected by heterozygous change-of-function missense mutations, *ZRSR2* is subjected to nonsense and frameshift mutations, which often result in loss of function.

Mutations of functionally conserved components of the spliceosome, such as U1 snRNA, change the splicing profile of multiple cancer driver genes by creating novel splice junctions resulting from impaired 5′ splice site recognition (Shuai et al. 2019). In fact, the g.3A > C mutation of U1 is associated with an unfavorable prognosis in patients with CLL. Interestingly, the U1 mutation seems to be mutually exclusive with *SF3B1* mutations in CLL. Although both mutations in CLL induce global splicing alterations, AS events are not shared between them: while CLL with U1 mutations tend to promote intron retention and reduce exon skipping events, *SF3B1* mutant cells display the reversed trend (Shuai et al. 2019).

Intriguingly, recent evidence indicates that SF mutations in myeloid malignancies promote the formation of R-loops, nucleic acid structures composed of RNA–DNA hybrids and an associated single-stranded DNA, thus leading to DNA damage and ATR-Chk1-pathway-mediated replication stress response (Chen et al. 2018; Singh et al. 2020). Therefore, R-loop-induced DNA damage might contribute to deleterious mutations in hematopoietic progenitor cells and aberrant cell proliferation. However, the role of R-loop formation in SF-mutated cancer cell biology seems to be much more complex. Notably, R-loops are involved in various physiological processes, including regulation of gene expression, chromatin structure, as well as DNA replication and DNA damage repair (Gambelli et al. 2023). Nevertheless, since R-loops were identified to play a key role in the maintenance of genomic integrity, and persistent abundance of DNA:RNA hybrid formation was linked to genome instability, further studies are needed to address this issue with respect to leukemogenesis. However, due to remarkable challenges in the mapping of R-loops in primary human cells, no reports regarding R-loop abundance and localization in malignant versus normal hematopoietic cells collected from patients have been reported to date. Emerging data indicate that the activity of transcript maturation and SFs is associated with R-loop management. Indeed, SRSF1 was found to inhibit R-loop formation during RNA polymerase II (RNAPII)-dependent transcription by interaction with single stranded RNA (Paz et al. 2021). Interestingly, SF-mutated cancers exhibit alterations in RNAPII processivity, which is related to R-loop generation during transcription. The formation of mutagenic R-loops along gene bodies may result from decreased rate of transcription elongation (Boddu et al. 2024). Recent study by Boddu et al. (2024) revealed that mutations in *SF3B1* reduce co-transcriptional splicing efficiency as well as transcription elongation rate, thus impairing splicing assembly. As a result of disrupted spliceosome assembly, the elongation rate of RNAPII is reduced and RNAPII density at promoters is decreased, leading to replication stress and chromatin landscape reorganization. These findings suggest that SF-mutated malignancies are diseases of mRNA processing and transcription abundance, not solely AS events.

### SF3B1

SF3B1, an essential component of the U2 snRNP, is the most frequently mutated SF across hematological malignancies. A key function of *SF3B1* in spliceosome assembly is to stabilize a duplex between U2 snRNA and a consensus BPS with the use of its C-terminal HEAT domain. Of note, in cancers, *SF3B1* mutations occur in consecutive repeats within the N-terminus of its HEAT domain (Maji et al. 2019). It is expected that functional effects of *SF3B1* mutations are associated with dysregulation of activity of regulatory networks due to AS of target genes. Different studies have reported changes in numerous cellular pathways, including MYC and NOTCH1 signaling, B-cell receptor signaling, DNA damage response, and telomere maintenance, in the leukemic *SF3B1* mutant samples (Liu et al. 2020; Wang et al. 2016; Yin et al. 2019). Nevertheless, the exact mechanisms underlying the aberrant regulation of signaling pathways in samples with mutated *SF3B1* have remained a mystery.

The most common *SF3B1* mutation among hematologic malignancies is the K700E substitution. The *SF3B1* K700 and R625 substitutions tend to use cryptic 3′ splice sites through increased expression of transcripts interacting with aberrant BPS (Darman et al. 2015; Canbezdi et al. 2021). This *SF3B1* mutation-induced splicing event generates many aberrant transcripts harboring PTC, which induces NMD (Darman et al. 2015). To date, NMD-mediated downregulation of several genes involved in cancer biology has been reported in samples with *SF3B1* mutation. For instance, *SF3B1* mutations promote aberrant 3′ splice site recognition of *MAP3K7* and PP2A phosphatase subunit *PPP2R5A*, thus inducing NMD of these transcripts (Lee et al. 2018).

Interestingly, *SF3B1* mutations in MDS patients are highly associated with the presence of ring sideroblasts (RS) (Malcovati et al. 2015). The RNA-seq analysis of patients with MDS with RS unveiled the role of *SF3B1* mutation in AS of the iron transporter *ABCB7* (Dolatshad et al. 2016). Dolatshad et al. (2016) found that SF3B1-mediated AS promotes mitochondrial iron accumulation in MDS-RS samples via downregulation of *ABCB7* due to NMD of the aberrantly spliced mRNA transcript. In addition to dysregulated iron metabolism homeostasis, accumulating data indicate that *SF3B1* mutations results in impaired erythropoiesis, an inflammatory microenvironment, and R-loop formation in patients with MDS (Jiang et al. 2023). In the 5th edition of the World Health Organization (WHO) classification criteria for MDS, MDS with *SF3B1* mutations has been classified as an independent subtype. Notably, *SF3B1*-mutated MDS-RS is characterized with low risk of conversion to leukemia and improved overall survival. Identification of splicing events generated by *SF3B1* mutation provided valuable insights in the process of erythropoiesis, thus enabling the application of erythropoiesis-stimulating agents in the treatment of patients with MDS. The erythropoietic transforming growth factor beta-targeting luspatercept is well tolerated and effective drug in the treatment of anemia in individuals with low-risk MDS (Jiang et al. 2023). Therefore, it was approved for the treatment of transfusion-dependent low-risk MDS with RS and/or *SF3B1* mutations.

It is important to note, that *SF3B1* mutations alter splicing patterns with numerous AS events. Despite the high incidence of 3′ splice site alterations in *SF3B1* mutant cells, a small but global reduction of intron-retaining variants was reported to be the most frequent aberrant splicing event in MDS samples with *SF3B1* mutation (Shiozawa et al. 2018). Although the functional relevance of the *SF3B1* mutation-induced splicing events in tumor cells is not well defined, their effects might serve as a source of neoantigens for the development of personalized vaccines or adoptive cell-based therapies (Schischlik et al. 2019).

### U2AF

The U2AF heterodimer plays a crucial role in the functional 3′ splice site recognition via base pairing with distinct splicing signals at the 3′ end of an intron. Both subunits of the complex—U2AF1 and U2AF2 (or U2AF35 and U2AF65, respectively) are recurrently mutated in hematologic malignancies, it is however unclear how these mutations impact the disease progress. To date, *U2AF1/2* mutations were reported to alter the splicing of genes involved in DNA damage response (*ATR*), apoptosis (*CASP8*), innate immune pathways (*IRAK4*), and DNA methylation (*DNMT3B*, *ASXL1*) (Ilagan et al. 2015; Smith et al. 2019).

In physical conditions, U2AF1 binds to the consensus AG dinucleotide at the 3′ end of an intron, while U2AF2 recognizes the polypyrimidine tract. To date, distinct mechanistic consequences of *U2AF* mutations have been described. Genome-wide studies of U2AF-RNA interactions showed that *U2AF* mutations in hematopoietic cells are associated with changed cassette exon usage (Ilagan et al. 2015). Interestingly, the mutant *U2AF1*-driven AS was found to result in different 3′ splice site motif selection in an allele-specific manner (Ilagan et al. 2015).

Furthermore, the study by Shirai et al. (Shirai et al. 2015) showed aberrant hematopoiesis and changed splicing patterns in hematopoietic progenitor cells in mice expressing mutant *U2AF1*. Notably, *U2AF1* mutation was associated with dysregulated splicing of numerous genes frequently affected by loss of function mutations in neoplasia, including MDS, such as *BCOR*, as well as genes involved in RNA processing and ribosome biogenesis (Shirai et al. 2015).

Recently, Park et al. (2016) revealed the oncogenic activity of mutant *U2AF35*. They found that *U2AF35*-transformed cells produce an abnormally translated isoform of autophagy-related factor 7 (*ATG7*), since mutant *U2AF35*^S34F^ promotes the selection of a distal poly(A) site in *ATG7* transcript. As a result of inefficient translation of *ATG7*, the expression of this protein is significantly decreased, thus impairing autophagy and promoting transformation. Moreover, Yip et al. (2017) showed aberrant erythroid and granulomonocytic differentiation in human hematopoietic progenitors with *U2AF1*^S34F^ mutation, which tend to be associated with the induction of differential splicing of genes encoding an H2A histone variant (*H2AFY*) and serine/threonine kinase receptor-associated protein (*STRAP*). Interestingly, *U2AF1*^S34F^ mutation changes the non-canonical function of U2AF1 in negative regulation of translation by altering the direct binding of the SF to 5′-UTR near the start codon, thus promoting the expression of chemokine IL8 (Palangat et al. 2019). Importantly, elevated levels of IL8 trigger inflammatory processes and cancer progression. Regarding hematologic malignancies, an increase of IL8 in human bone marrow cells is highly related to relapsed/refractory AML (Schinke et al. 2015).

U2AF2 mutations mainly cluster within the two central RNA recognition motifs, which play a key role in polypyrimidine tract recognition. In contrast to the more common *U2AF1* aberrations, characterization of leukemia-relevant *U2AF2* mutations and their functional consequences are lacking. Nevertheless, a recent study indicated the capability of *U2AF2* mutations to dysregulate gene expression profiles, thereby contributing to neoplastic transformation (Maji et al. 2020). Smith et al. (2019) reported that mutations in *U2AF2* induce differential splicing of interleukin-1 receptor-associated kinase 4 (*IRAK4*) in AML and MDS, which leads to the accumulation of longer transcript that retains exon 4, called IRAK4-long (*IRAK4-L*). IRAK4-L confers a growth advantage to leukemic cells via activation of NF-κB as well as mitogen-activated protein kinase (MAPK) through assembling with MyD88. Of note, the expression of oncogenic IRAK4-L correlates with unfavorable prognosis in patients with AML (Smith et al. 2019).

## Splicing-related mutations in hematological malignancies: auxiliary splicing factors

As mentioned above, spliceosome assembly is modulated by numerous splicing regulators, including SR proteins, hnRNPs, and proteins with RNA-binding motifs (RBMs). Basically, the activity of these splicing regulatory factors is determined by the nature of neighboring pre-mRNA sequences. As the auxiliary SFs utilize specific nucleotide sequences in a position-dependent manner, mutations affecting these RNA-binding proteins might promote neoplastic transformation due to splice-site disruption, and in turn, differential AS of cancer-related genes (Jayasinghe et al. 2018).

### SRSF2

Recently, it was reported that mutation in serine/arginine splicing factor 2 (*SRSF2*^P95H^) change its RNA-binding specificity, thus altering the splicing of several genes associated with leukemogenesis and MDS (Liang et al. 2018). In physical conditions, SRSF2 contributes to exon recognition by interacting with ESE motifs within pre-mRNA. It was shown that mutated *SRSF2* directly impairs hematopoietic cell differentiation due to changed exon inclusion resulting from an aberrant affinity for ESEs (Kim et al. 2015). Furthermore, mutated *SRSF2* was found to dysregulate splicing of a key transcriptional regulator that has recently been implicated in the pathogenesis of myeloid malignancies – enhancer of zeste homolog 2 (*EZH2*) (Kim et al. 2015). Intriguingly, *SRSF2* mutations and loss-of-function mutations in *EZH2* are mutually exclusive in patients with MDS (Kim et al. 2015).

Furthermore, *SRSF2*^PH95^ mutation contributes to enhanced activity of the NMD-inducing pathway in AML samples (Rahman et al. 2020). For instance, a differential splicing pattern in *IDH2* and *SRSF2* double-mutant cells was characterized by increased intron retention, which contributed to NMD-related reduced expression of integrator subunit 3 (*INTS3*), and thereby malignant transformation (Yoshimi et al. 2019). Nevertheless, the functional effect of individual *SRSF2* mutations needs further investigation, as each mutation results in a unique splicing profile in leukemic cells (Pangallo et al. 2020).

### ZRSR2

There are two types of machinery catalyzing the RNA-splicing: U2-dependent spliceosome, which recognizes the majority of introns (U2-type intron), and U12-dependent spliceosome, which removes highly conserved U12-type introns. Due to distinct splice sites and branchpoints, U12-type introns are removed by separate splicing machinery, called the minor spliceosome. U12-type introns are a small subset (< 0.5%) of all introns and are often found in genes that have been attributed a crucial role in RNA processing and cell cycle regulation (Turunen et al. 2013). Although several genes with U12-type introns have been implicated in cancerogenesis, the functional consequences of their aberrant expression due to minor intron retention require further investigation.

One of the key components of the minor spliceosome assembly is *ZRSR2*, an RBP that recognizes the 3′ splice site of U12-type introns. Interestingly, mutations in the X-chromosome encoded *ZRSR2* are frequently found in male patients with MDS (Madan et al. 2015). Furthermore, mutations in *ZRSR2* are associated with an increased minor intron retention (Madan et al. 2015). Recently, Inoue et al. (2021) reported that dysregulation of a regulator of Ras-related GTPases *LZTR1* in MDS is frequently induced by aberrant minor intron excision caused by *ZRSR2* loss. Moreover, they found that an impaired minor intron splicing, induced by *ZRSR2* loss, improved hematopoietic stem cell self-renewal.

## Splicing-related mutations in hematological malignancies: other spliceosome components and *cis*-acting elements

Accumulating evidence indicates that proteins involved in the late stages of spliceosome formation and RNA processing might also be affected by mutations, thus contributing to hematological malignancies. Indeed, mutations in *PRPF8*, a gene encoding the most evolutionarily conserved spliceosomal protein, have been found in ~ 3% of patients with myeloid neoplasms (Kurtovic-Kozaric et al. 2015). Kurtovic-Kozaric et al. (2015) demonstrated that loss-of-function mutations in *PRPF8* result in global modulation of cassette exon usage. Furthermore, they reported that the *PRPF8*-induced missplicing defects lead to enhanced cellular proliferation, resulting in a distinct phenotype of aggressive MDS with increased RS. As confirmed in yeast models, *PRPF8* disturbs the second catalytic step of the spliceosome assembly due to its impaired proof-reading functions. These defects might be associated with increased lifetimes of nonfunctional spliceosomal complexes, which contribute to widespread aberrant splicing of genes, especially those involved in mitochondrial metabolism and hematopoiesis (Kurtovic-Kozaric et al. 2015).

Frameshift and nonsense mutations lead to a loss of function of another SF, *LUC7L2*. Although the function of LUC7L2 has not been yet determined, this SF was found to interact with components of the U1 and U2, as well as with other splicing regulators in the nucleus (Daniels et al. 2021). Reduced *LUC7L2* expression or mutation is associated with significantly shorter patients’ survival in myeloid malignancies (Hosono et al. 2014). It was reported that knockdown of *LUC7L2* dysregulates AS pattern due to aberrant 5′ splice site recognition (Daniels et al. 2021). Of note, loss of *LUC7L2* downregulates glycolytic genes, which might change cellular metabolism, thus contributing to disease pathogenesis (Daniels et al. 2021). However, the mechanistic role of *LUC7L2* and its abnormalities in RNA processing needs further investigation.

Recently, germline and somatic mutations of the DEAD-box helicase 41 gene (*DDX41*) have been found to promote the development of MDS and AML (Sébert et al. 2019; Badar and Chlon 2022). Myeloid neoplasms with *DDX41* mutation are characterized by long latency and high-risk disease at presentation with normal karyotype (Badar and Chlon 2022). Chlon et al. (2021) reported a disrupted snoRNA processing and ribosome activity that contribute to hematopoietic defects in biallelic *DDX41* mutant bone marrow cells. Interestingly, MDS patients with germline monoallelic frameshift *DDX41* mutations were found to subsequently acquire of a somatic *DDX41* variant in their other *DDX41* allele (Chlon et al. 2021). Moreover, *DDX41* mutations in MDS were found to be associated with the presence of *TP53* mutation (Quesada et al. 2019). Finally, a study by Mosler et al. (2021) shed a light on the mechanistic role of *DDX41* mutations in myeloid malignancies. They demonstrated that *DDX41* loss leads to the accumulation of co-transcriptional R-loops accompanied by replication stress, enhanced formation double-strand and DNA breaks and inflammatory response, which might contribute to the development of the disease.

More recently, a large-scale genomic study revealed recurrent mutations in *DAZAP1*, *EWSR1*, and *HNRNPH1*, thus evidencing that AS-regulating RBPs are commonly mutated also in patients with mantle cell lymphoma (MCL) (Pararajalingam et al. 2020). Furthermore, novel recurrent noncoding mutations affecting a single exon of *HNRNPH1* have been described. Functionally, HNRNPH1 comprises a hnRNP family of RBPs, which mediates transcription by repressing splicing. It was demonstrated that the specific mutation-induced AS of *HNRNPH1* promotes the expression of *HNRNPH1* variant, which escapes NMD, thereby disrupting the self-regulation of the protein expression in MCL (Pararajalingam et al. 2020). This *HNRNPH1* mutant-like splicing profile that favors the productive variant was reported to be associated with adverse outcomes in patients with MCL (Pararajalingam et al. 2020).

It has been suggested that somatic mutations directly affecting *cis*-acting elements might contribute to cancerogenesis due to either the introduction of new splicing regulatory elements or the disruption of existing ones. Furthermore, an analysis of over 3000 cancer exomes indicates that silent or synonymous mutations contribute to cancer, frequently through changes in splicing (Supek et al. 2014). The study demonstrated a synonymous mutation-induced gain of ESE motifs as well as the loss of ESS motifs in cancer cells. Of note, ESEs and ESSs are hypothesized to be more essential for exon definition in case of weak (nonconsensus) flanking splice sites. Supporting this notion, Supek et al. (2014) found weaker splice sites in exons of analyzed oncogene set, which harbored more synonymous mutations. It was demonstrated that both mutations creating ESEs and disrupting ESSs affected leukemia-related oncogenes, including *PDGFRA*, *EGFR*, *JAK3*, *GATA*, and *BCL6*, and tumor suppressor genes, such as *TP53* (Supek et al. 2014). Moreover, a study of exome data from > 1800 tumor samples identified ~ 900 somatic exonic mutations, which lead to aberrant splicing (Jung et al. 2015). Among these, at least 163 mutations were found to induce intron retention or exon skipping. Remarkably, tumor suppressor genes, such as *TP53* and *CDKN2A*, were significantly enriched in intron retention-causing mutations, which resulted in their inactivation due to NMD or truncated protein (Jung et al. 2015). Recently, a full-length differential transcript analysis of CLL samples demonstrated downregulation of intron retention in cells with *SF3B1*^K700E^ mutation (Tang et al. 2020). Moreover, Jayasinghe et al. (2018) identified over 1900 splice-site-creating mutations (SCMs) in > 8600 TGCA (The Cancer Genome Atlas) tumor samples, thereby unveiling novel splice sites in cancer-related genes, including *TP53* and *GATA3*. Interestingly, neoantigens induced by SCM were found to be more immunogenic in comparison to those derived from missense mutations, and thus might be considered as immunotherapy targets. Additionally, tumor cells with SCMs exhibited an increased expression of PD-L1 and high T cell immune response, suggesting the potential immunotherapy in these cases (Jayasinghe et al. 2018).

## Indirect regulation of splicing

Chromosomal rearrangements and altered activity of transcriptome machinery might also be associated with aberrant splicing regulation. For instance, Dvinge and Bradley (2015) found that increased intron retention in AML samples correlates with the presence of *RUNX1*, *IDH1*, and *IDH2* mutations. These results imply that aberrant DNA methylation driven by mutations in epigenetic regulators *IDH1* and *IDH2* can influence AS profile in leukemic cells through altered intron recognition. However, previous reports showed that AS could be affected by changed DNA methylation resulting from differential CTCF binding, as exons exhibit an increased methylation level relative to intronic sequences (Gelfman et al. 2013).

A recent study performed a transcriptome-wide analysis of AML samples, thus identifying a common overlap of mutations in *IDH2* and *SRSF2*, which together promote leukemic transformation (Jayasinghe et al. 2018). Interestingly, while mutations in either *IDH2* or *SRSF2* induce splicing changes, co-occurrence of *SRSF2* and *IDH2* mutations cause more profound splicing aberrations compared to the samples with either mutation alone. Indeed, in vivo study indicated that co-expression of mutant *SRSF2* and *IDH2* led to the development of lethal MDS with proliferative features and enhanced self-renewal of the cells (Jayasinghe et al. 2018).

Furthermore, Huang et al. (2022) reported that the loss of transcription factor *RUNX1* affects the gene expression profile in MDS samples and the coexistence of *SRSF2*^P95H^ mutation further perturbs the transcriptional regulation of genes involved in several processes relevant to blood malignancies, such as cell proliferation and inflammatory response, as well as genes recurrently mutated in hematological disorders, including *ATM* and *EZH2*.

## Cellular implications of aberrant splicing

To date, numerous alternative variants have been associated with disrupted cell metabolism and cancerogenesis. For instance, differentially spliced isoforms of apoptosis-related genes can generate proteins with opposite functions, thus affecting apoptotic regulation (Table 2).

**Table 2:** Exon/intron usage and biological function of alternatively spliced genes involved in selected biological pathways

Apoptosis					
Gene	AS event	Splicing regulator	Produced isoforms	Biological function	References
*BCL-X*	Alternative 5′ splice site in (exon 2)	SF3B1, SRSF1, SRSF2, RBM4, RBM10	*BCL-XS*	Pro-apoptotic	Inoue et al. (2014); Bielli et al. (2014); Wang et al. (2014); Stevens and Oltean (2019)
			*BCL-XL*	Anti-apoptotic	
*MCL-1*	Cassette exons (exon 2)	SF3B1, SRSF1	*MCL-1S*	Pro-apoptotic	Necochea-Campion et al. (2015); Pearson et al. (2020); Moore et al. (2010)
			*MCL-1L*	Anti-apoptotic	
*BIM*	Mutually exclusive exons 3 and 4	SRSF1, PTBP1	*BIM*^+exon3^	Pro-apoptotic	Juan et al. (2014); Ko et al. (2016)
			*BIM*^+exon4^	Anti-apoptotic	
*FAS*	Exon skipping (exon 6)	SPF45, TIA-1, PTB, RBM10, SRSF6	*Fas*	Pro-apoptotic	Inoue et al. (2014); Izquierdo et al. (2005); Choi et al. (2022)
			*Fas*^−exon6^	Anti-apoptotic	
*Survivin*	Exon skipping (exon 3, exon 2B)	N/A	Survivin 2 $\beta$ and 2 $\alpha$	Pro-apoptotic	Wagner et al. (2006); Végran et al. (2013)
			Survivin $\Delta$ Ex3 and 3 $\beta$	Anti-apoptotic	
*c-FLIP*	Exon skipping (exon 7)	RBM5, RBM10	*c-FLIP*_*L*_^−exon7^	Pro-apoptotic	Inoue et al. (2014); Bielli et al. (2014); Wang et al. (2014); Stevens and Oltean (2019); Necochea-Campion et al. (2015); Pearson et al. (2020); Moore et al. (2010); Juan et al. (2014); Ko et al. (2016); Izquierdo et al. (2005); Choi et al. (2022); Wagner et al. (2006); Végran et al. (2013); Mclornan et al. (2013)
			*c-FLIP*_*S*_^+exon7^	Anti-apoptotic	
Cell signaling					
*CASP8*	Cassette exons 6 and 7	SRSF2 U2AF1	*CASP8*^*−*exon6and7^	Promotes NF-κB signaling	Lee et al. (2018); Ilagan et al. (2015)
			*CASP8-L*	Anti-apoptotic	
*MAP3K7*	Aberrant 3′ splice site (exon 5)	SF3B1	Out-of-frame *MAP3K7* transcript that undergoes NMD	Promotes NF-κB signaling	Lee et al. (2018)
			*MAP3K7*	Regulation of NF-κB, JNK and MAPK pathways	
*KLF6*	Alternative 5′ splice sites (exon 3)	SRSF1	Wild-type *KLF6*	Tumor suppressor	Hu et al. (2021); Muñoz et al. (2012)
			*KLF6-SV1*	Tumor cell proliferation, invasion, and metastasis	
Cell metabolism					
*PKM*	Mutually exclusive exons 9 and 10	PTBP1	*PKM1*^+exon9^	Glycolysis regulation (constitutively active isoform)	Wang et al. (2019b); Huang et al. (2021)
			*PKM2*^+exon10^	Glycolysis regulation (allosterically regulated isoform)	

Overexpression of BCL-XL as well as other anti-apoptotic proteins have been reported to be correlated with chemotherapy resistance in various cancer types, including hematological malignancies (Yoshimi et al. 2019; Necochea-Campion et al. 2016; Zhang et al. 2020). Nevertheless, splicing modulators can effectively regulate AS of apoptotic proteins to favor leukemic cell sensitization to therapeutic agents. For instance, Moore et al. (2010) revealed that drug-induced mitotic arrest results in the downregulation of the *SRSF1*, which in turn promotes the synthesis of pro-apoptotic *BCL-XS* and *MCL-1S* isoforms.

Recently, it was reported that enhanced production of the oncogenic splicing isoform of the Kruppel-Like Factor 6 (*KLF6-SV1*) is significantly associated with the proliferation of cancer cells and might play an important role in regulating apoptosis (Hu et al. 2021). In vitro and in vivo studies showed a KLF6-SV1-mediated anti-apoptotic effect of T cells on CLL cells (Kokhaei et al. 2018).

To date, cancer cells were found to express numerous alternative splice isoforms of the anti-apoptotic protein survivin with various levels of association with distinct prognostic features and drug resistance (Wagner et al. 2006; Végran et al. 2013; Moore et al. 2014). Indeed, it was reported that increased expression of a particular survivin splice variant (*survivin-*$\Delta$* Ex3*) is significantly associated with unfavorable survival outcomes in pediatric individuals with AML, while high expression of survivin-2b was found to be associated with better survival in adult patients with AML (Wagner et al. 2006).

Apoptotic signaling might be also affected by differential splicing of *c-FLIP*. The study by McIornan et al. (2013) demonstrated that increased expression of the longer variant c-FLIP_L_ is associated with significantly shorter 3-year overall survival in adult AML patients. Nevertheless, both c-FLIP_L_ and c-FLIP_S_ variants might contribute to cancer progression, as they influence cytoprotective and pro-survival pathways, such as AKT, ERK and NF-κB (Safa 2012).

Evidence from the past decade identified *NOTCH2* and *FLT3* differential splicing as a common event in AML (Adamia et al. 2014b). *FLT3* splicing results in the production of isoforms that affect key downstream signaling targets, such as AKT and STAT, and thereby promote the transduction of pro-survival and proliferative signals. Altered *NOTCH2* and *FLT3* splice variants are generated upon complete or partial exon skipping and selection of cryptic splice sites. Furthermore, it was reported that specific *FLT3* isoforms are overexpressed at diagnosis and relapse, but not elevated during remission in patients with AML. This study also revealed the association between *NOTCH2-Va* splice variant expression and unfavorable outcomes, especially for individuals with an intermediate-risk cytogenetic profile. Of note, aberrant splicing in AML cells is independent of the presence of any splicing factor mutation (Adamia et al. 2014b).

Although *SF3B1* or *SRSF2* mutations have a distinct impact on AS patterns, they promote NF-κB signaling pathway activation (Lee et al. 2018). In the case of *SF3B1*, the NF-κB axis is induced by aberrant 3′ splice site selection in *MAP3K7*, while in *SRSF2* mutant cells NF-κB activity is mediated by skipping of a cassette exon, which leads to the generation of a C-terminal truncated variant of caspase 8. The *Sf3b1*^K700E^-mediated mis-splicing of *MAP3K7* was reported to affect the NF-κB pathway activation in MDS samples. The truncated CASP8 isoform was found to hyperactivate NF-κB signaling in *SRSF2*-mutated patients with AML and CMML. Interestingly, despite the mis-spliced isoform of caspase 8 promotes NF-κB signaling, it has no effect on cell death. This observation was recently confirmed in a study, which revealed that mutations in *SF3B1* or *SRSF2* are mutually exclusive due to both synthetic lethal interactions and convergent effects on the activation of innate immune signaling (Lee et al. 2018).

Recently, Wang et al. (2019b) reported that high expression of the pyruvate kinase M2 (PKM2) in *NPM1*-mutated AML mediates autophagic activation and is associated with unfavorable clinical outcomes. Moreover, evidence presented by Huang et al. (2021) identified higher plasma levels of differentially spliced *PKM2* isoform in AML and ALL, which negatively correlated with disease prognosis. Notably, PKM2 overexpression contributes to leukemic cell proliferation, differentiation and drug resistance via both aerobic glycolysis and non-metabolic pathways [reviewed in Yang et al. (2021)].

## Conclusions

Previous studies indicate that aberrant splicing is a common event in leukemia development and progression. However, the function of the spliceosome is complex as the outcome of AS deregulation differs between various hematological malignancies. Moreover, most of the experimental approaches regarding characterization of mechanisms underlying splicing-related aberrations (mutations, changed expression or activity of a specific splicing-related genes) involve the use of human cancer cell lines, not primary cell cultures. Thus, additional studies are needed to provide further insights into the mechanistic consequences of distinct splicing changes in the cellular context of patients with different blood disorders. Another issue regarding studies on AS is limited evidence for alternative proteins in proteomics analyses, as it is still not clear how many alternatively spliced isoforms produce functionally relevant protein. Taking into consideration that the majority of alternative exons are evolving neutrally (Tress et al. 2017), it seems crucial to determine functions of specific isoforms produced from alternatively spliced mRNAs, as it could help to unveil the real impact of distinct somatic mutations observed in tumors. For instance, application of machine learning algorithms that uses proteomics evidence would be of great value to extract datasets with less noise and enriched in biologically relevant isoforms (Pozo et al. 2021). Such tools will be help to understand the pathogenic effects of particular splicing-related gene mutation on splice isoform, and, in turn, evaluate how observed splicing events relate to a patient’s outcome.

## Data Availability

Not applicable.

## Abbreviations

AS: Alternative splicing
SF: Splicing factor
PTC: Premature termination codon
NMD: Nonsense-mediated decay
UTR: Untranslated region
snRNP: Small nuclear ribonucleoprotein particle
BPS: Branchpoint sequence
SF1: Splicing factor 1
UAAF2: U2 auxiliary factor 2
ESE: Exonic splicing enhancer
ESS: Exonic splicing silencer
ISE: Intronic splicing silencer
ISS: Splicing silencer
RBP: RNA binding protein
hnRNP: Heterogenous nuclear RNP
DLBCL: Diffuse large B-cell lymphoma
AML: Acute myeloid leukemia
MDS: Myelodysplastic syndrome
CLL: Chronic lymphocytic leukemia
RS: Ring sideroblasts
U2AF35: Autophagy-related factor 7
STRAP: Serine/threonine kinase receptor-associated protein
IRAK4: Interleukin-1 receptor-associated kinase 4
MAPK: Mitogen-activated protein kinase
RBM: RNA binding motif
SRSF2: Serine/arginine splicing factor 2
EZH2: Zeste homolog 2
INTS3: Integrator subunit 3
DDX41: DEAD-box helicase 41 gene
MCL: Mantle cell lymphoma
SCM: Splice-site-creating mutation
KLF6-SV1: Kruppel-Like Factor 6
